# Crystal structure of 4-(meth­oxy­carbon­yl)phenyl­boronic acid

**DOI:** 10.1107/S2056989015015923

**Published:** 2015-09-12

**Authors:** Keith J. Flanagan, Mathias O. Senge

**Affiliations:** a School of Chemistry, SFI Tetrapyrrole Laboratory, Trinity Biomedical Sciences Institute, 152-160 Pearse Street, Trinity College Dublin, The University of Dublin, Dublin 2, Ireland

**Keywords:** crystal structure, boronic acid, meth­oxy­carbon­yl, ester, protecting groups, Suzuki coupling, hydrogen bonding, inversion dimers, π–π inter­actions.

## Abstract

In the title compound, C_8_H_9_BO_4_, the meth­oxy­carbonyl group is rotated out of the plane of the benzene ring by 7.70 (6)°. In the crystal, mol­ecules are linked *via* pairs of O—H⋯O hydrogen bonds, involving the boronic acid OH groups, forming inversion dimers. The dimers are linked *via* O—H⋯O hydrogen bonds, involving a boronic acid OH group and the carbonyl O atom, forming undulating sheets parallel to (10-2). Within the sheets there are also C—H⋯O hydrogen bonds present, also involving the carbonyl O atom. The sheets are linked *via* C—H⋯π and offset face-to-face π-inter­actions between inversion-related mol­ecules [inter-centroid distance = 3.7843 (16) Å, inter­planar distance = 3.3427 (4) Å and offset = 1.744 Å], forming a three-dimensional structure.

## Abstract 2

In the crystal of the title compound, mol­ecules are linked *via* O—H⋯O and C—H⋯O hydrogen bonds, forming undulating sheets parallel to (10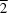). The sheets are linked *via* C—H⋯π and offset face-to-face π-inter­actions, between inversion-related mol­ecules [inter-centroid distance = 3.7843 (16) Å], forming a three-dimensional structure.

## Chemical context   

Boronic acids have been widely studied, mainly due to their roles in coupling reactions such as Suzuki (Suzuki, 2011 ▸), Chan–Lam (Lam *et al.*, 2000 ▸) and Liebeskind–Srogl (Liebeskind & Srogl, 2000 ▸). Complexes of boronic acids are well known, and many examples have been structurally characterized (*e.g.*, Roşca *et al.*, 2012 ▸; Filthaus *et al.*, 2008 ▸; Cyrański *et al.*, 2008 ▸; Rettig & Trotter, 1977 ▸). Many examples exist of similar compounds such as 2-methyl­imidazolium (4-carb­oxy­benzene)(2-methyl­imidazol­yl)boronate monohydrate (Aakeröy *et al.*, 2005 ▸) and 4-carb­oxy­phenyl­boronic acid (SeethaLekshmi & Pedireddi, 2007 ▸). However, no examples of meth­oxy-protected derivatives have been published to date. We report herein on the crystal structure of the title compound, the 4-(meth­oxy­carbon­yl) derivative of phenyl­boronic acid.
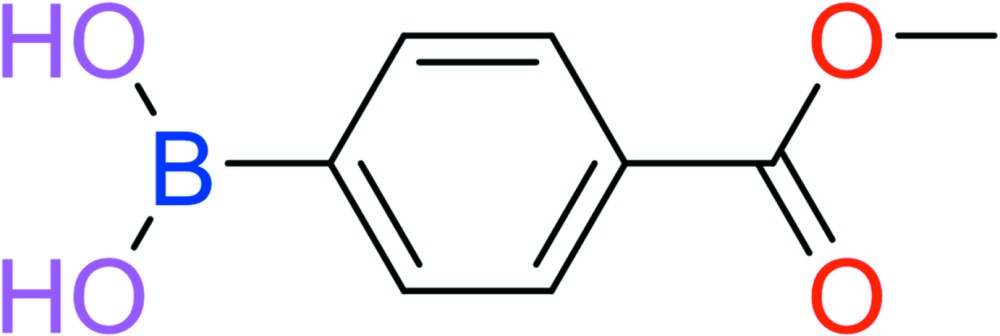


## Structural commentary   

**Figure 1:**
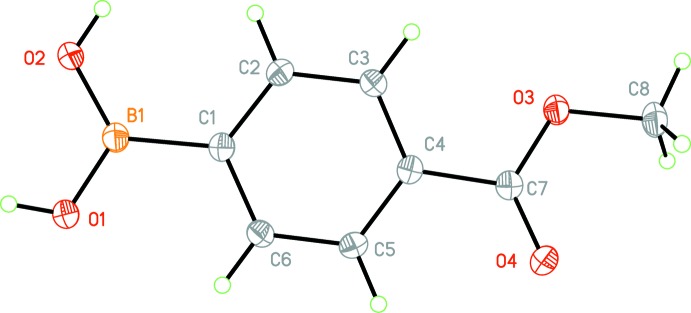
The mol­ecular structure of the title compound, showing the atom labelling. Displacement ellipsoids are drawn at the 50% probability level.

The title mol­ecule, Fig. 1 ▸, is almost completely planar with the meth­oxy­carbonyl group inclined to the benzene ring by 7.70 (6)°. The angle around atom B1, O1—B1—O2 is 118.16 (9)°, very close to the ideal value of 120°. The bond lengths and angles are similar to those reported for 4-carb­oxy­phenyl­boronic acid derivatives (SeethaLekshmi & Pedireddi, 2007 ▸) in which the carb­oxy­lic group is rotated from the plane of the benzene ring by *ca* 13.83–26.44°, and the O—B—O bond angles are in the range of ca 118.1–122.5°. Aakeröy *et al.* (2005 ▸) reported the structures of 4-acetyl­pyridine oxime 4-carb­oxy­benzene­boronate dihydrate and 4-acetyl­pyridine oxime 4-carb­oxy­benzene­boronate dihydrate in which the 4-carb­oxy groups are inclined to the benzene ring by *ca* 10.45–14.08°, close to the value observed for the meth­oxy­carbonyl group in the title compound.

## Supra­molecular features   

**Figure 2:**
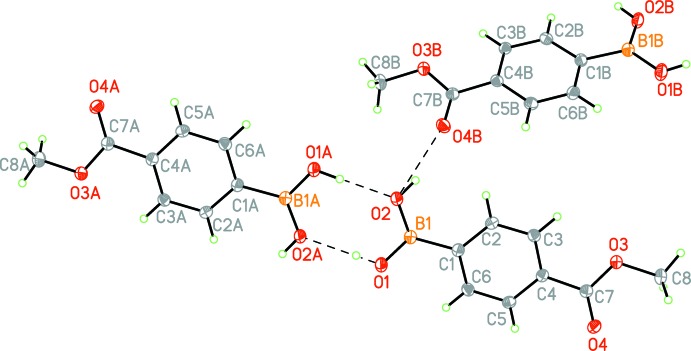
Hydrogen bonding (dashed lines) in the crystal of the title compound [see Table 1 ▸ for details; symmetry codes: (A) −*x* + 2, −*y* + 1, −*z* + 2; (B) −*x* + 1, *y* − 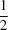, −*z* + 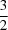]. Displacement ellipsoids are drawn at the 50% probability level.

In the crystal of the title compound, there are hydrogen bonds between the carbonyl atom O4 and the hy­droxy group O2–H2*A* of the boronic acid and atom O2 of the boronic acid with a *D⋯* *A* distance of 2.753 (1) Å (Fig. 2 ▸ and Table 1 ▸). The hy­droxy group O1*–*-H1*A* of the boronic acid is in an inversion-related hydrogen-bonded network with the oxygen O2 of the boronic acid at a distance of 2.762 (1) Å (Fig. 2 ▸ and Table 1 ▸).

The presence of the meth­oxy group on the carbonyl removes hydrogen-bond donation of the carb­oxy­lic acid seen in related structures. Atom C8 creates a shield around atom O3, removing its ability to participate in hydrogen bonding due to steric effects. It is noteworthy that the meth­oxy protecting group is small compared to other protecting groups and therefore exhibits no steric effects on the hydrogen-bonding capabilities to atom O4 in this structure, as seen in other examples. As exemplified by the work of Lemmerer (2012 ▸), most literature examples exhibit an almost exclusive head-to-tail hydrogen-bonding network between the boronic and carb­oxy­lic acids whereas the title compound exhibits exclusively head-to-head hydrogen-bonding inter­actions with the boronic acid subunit. This is due to the steric effects and removal of hydrogen-donating abilities in the meth­oxy­carbonyl subunit (Fig. 2 ▸). The group can still act as a hydrogen acceptor, as shown in the packing diagram (Fig. 2 ▸). Atom O4 can accept H atoms from the hy­droxy group O2—H2*A* to create an offset face-to-face overlap in the packing unit. Yang *et al.* (2005 ▸) published a structure of the boronic ester deriv­ative of the title compound. This structure showed similar effects, however no hydrogen bonds were visible in the reported structure.

**Figure 3:**
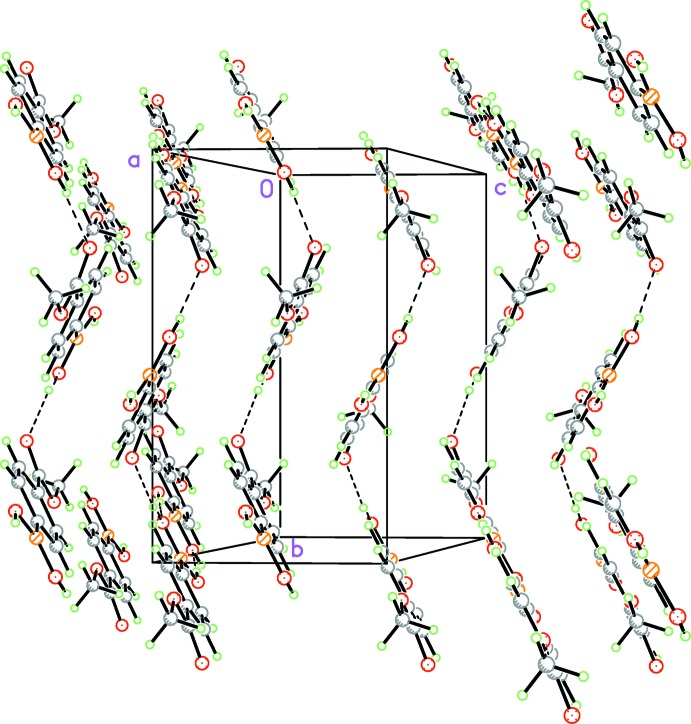
View of the π-aggregated structure as viewed approximately along the *a* axis.

**Figure 4:**
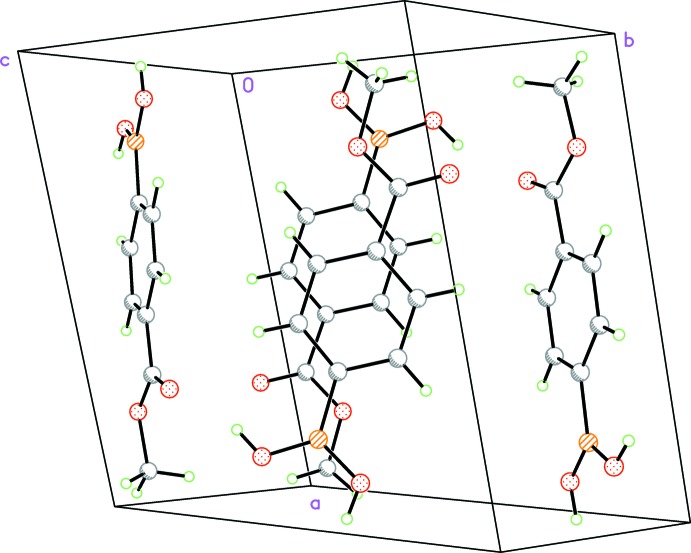
A view approximately along the *b* axis, showing the offset face-to-face π-inter­actions involving inversion-related mol­ecules.

**Figure 5:**
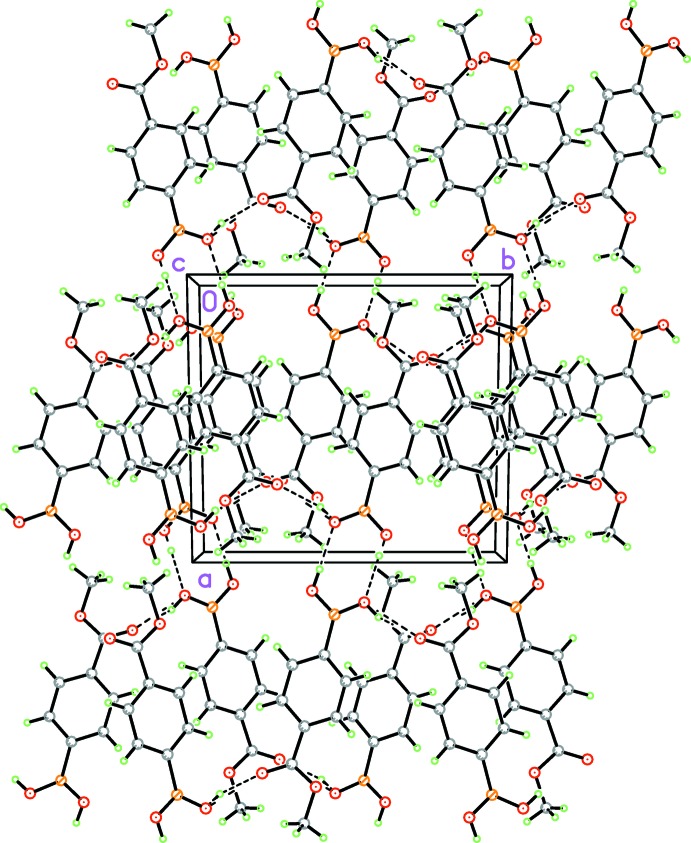
Crystal packing diagram of the title compound viewed along the *c* axis, showing the offset face-to-face π-inter­actions involving inversion-related mol­ecules (dashed lines; see Table 1 ▸ for details).

Hydrogen bonding and π-stacking within the unit cell forms a strong set of dimeric pairs. This can be easily observed in Fig. 3 ▸. These dimeric pairs line up to form a zigzag stacking pattern with a consistent spacing throughout the unit cell. This is aided by the hydrogen bond between O4 and O2—H2*A* [2.753 (1) Å] and a close contact between atom O4 and the hydrogen on atom C2 at a distance of 2.59 Å. The unit cell consists of four mol­ecular units which form π-aggregated pairs in a head-to-tail fashion and are stabilized through offset face-to-face π-inter­actions [inter-centroid distance = 3.7843 (16) Å; inter-planar distance = 3.3427 (4) Å, offset = 1.744 Å]; see Fig. 4 ▸. The hy­droxy group O1—H1*A* of the boronic acid is in an inversion-related hydrogen-bonded network with the oxygen O2 of the boronic acid at a distance of 2.762 (1) Å, forming a head-to-head hydrogen-bonded network with adjacent mol­ecules (Fig. 5 ▸). There are also C—H⋯π inter­actions (Table 1 ▸) present between the undulating sheets parallel to (10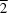). The sum of all of these inter­molecular inter­actions leads to the formation of a three-dimensional structure.

## Database survey   

A search of the Cambridge Structural Database (Version 5.36, update Nov. 2014; Groom & Allen, 2014 ▸) gave fourteen hits for free carb­oxy­lic acid derivatives of the title compound and one for the boronic ester. Soundararajan *et al.* (1993 ▸) published a structure of 4-carb­oxy-2-nitro­benzene­boronic acid. The carb­oxy­lic group deviated from the mean plane with an angle of *ca* 5.84° and the O—B—O bond angle was *ca* 119.88°. Aakeröy *et al.* (2004 ▸) reported the structure of a 4-carb­oxy­benzene­boronic acid 4,4′-bi­pyridine derivative with the carb­oxy­lic group being rotated from the plane by *ca* 4.20° and an O—B—O bond angle of *ca* 118.09°. They also reported the structures of 2-methyl­imidazolium(4-carb­oxy­benzene)(2-methyl­imidazol­yl)boronate monohydrate, tris­(4-(di­methyl­amino)­pyridinium) bis­(4-(di­methyl­amino)­pyridine) tris­(4-carb­oxy­benzene­boronate) trihydrate and 4-acetyl­pyridine oxime 4-carb­oxy­benzene­boronate dihydrate which presented out-of-plane tilt angles of *ca* 10.45–27.74° and O—B—O bond angles of *ca* 114.23–124.94° (Aakeröy *et al.*, 2005 ▸).

SeethaLekshmi & Pedireddi (2006 ▸) reported on a selection of carboxyl­ato­phenyl­boronic acid derivatives, hexa­aqua-*M*(II) bis­(4-carboxyl­ato­phenyl­boronic acid) tetra­hydrate, where *M* is nickel, manganese or cobalt. These structures where similar to the title compound and exhibited similar characteristics for the O—B—O bond angle and the out-of-plane tilt of the carboxyl acid group compared to the title compound. The carb­oxy­lic group deviated from the plane with an angle of *ca* 3.40–4.53° and the O—B—O bond angles were in the range of *ca* 121.43–122.18° (SeethaLekshmi & Pedireddi, 2006 ▸). They also published a selection of 4-carb­oxy­phenyl­boronic acids including the monohydrate and the hydrate derivatives of this compound. The carb­oxy­lic group deviated from the mean plane with an angle of *ca* 13.83–26.44° and the O—B—O bond angles were in the range of *ca* 118.08–122.50° (SeethaLekshmi & Pedireddi, 2007 ▸).

The structure of bis­(8-chloro-1-methyl-6-phenyl-4*H*-[1,2,4]triazolo[4,3-a][1,4]benzodiazepine) 4-(di­hydroxy­bor­yl)benzoic acid monohydrate exhibited a tilt angle of *ca* 2.14° for the carb­oxy­lic group and an O—B—O bond angle of *ca* 126.53° (Varughese *et al.*, 2011 ▸). Likewise, the relevant values in the structure of a cyclo­penta­naminium 4-(di­hydroxy­bor­yl)benzoate trihydrate were *ca* 29.67 and 126.53°, respectively (Lemmerer, 2012 ▸). Finally, methyl 4-(4,4,5,5-tetra­methyl-1,3,2-dioxaborolan-2-yl)benzoate showed similar features to the title compound with a meth­oxy­carbonyl deviation from the ring plane of *ca* 4.97° (Yang *et al.*, 2005 ▸).

## Synthesis and crystallization   

The compound was purchased from Alfa Aesar and was purified with silica gel column chromatography using CH_2_Cl_2_:MeOH (19:1). The compound was then crystallized from a solution of 1% MeOH in CH_2_Cl_2_ layered with hexane to give a single crystal suitable for X-ray diffraction.

## Refinement   

Crystal data, data collection and structure refinement details are summarized in Table 2 ▸. The donor OH H atoms were located in a difference Fourier map and freely refined. The C-bound H atoms were placed in their expected calculated positions and refined as riding: C—H = 0.95–0.98 Å with *U*_iso_(H) = 1.5*U*_eq_(C) for methyl H atoms and 1.2*U*_iso_(C) for other H atoms.

## Supplementary Material

Crystal structure: contains datablock(s) I, publication_text. DOI: 10.1107/S2056989015015923/su5188sup1.cif

Structure factors: contains datablock(s) I. DOI: 10.1107/S2056989015015923/su5188Isup2.hkl

Supporting information file. DOI: 10.1107/S2056989015015923/su5188Isup3.cml

CCDC reference: 1416885

Additional supporting information:  crystallographic information; 3D view; checkCIF report

## Figures and Tables

**Table 1:** Hydrogen-bond geometry (, ) *Cg* is the centroid of ring C1C6.

*D*H*A*	*D*H	H*A*	*D* *A*	*D*H*A*
O1H1*A*O2^i^	0.86(2)	1.90(2)	2.762(1)	178.4(18)
O2H2*A*O4^ii^	0.84(2)	1.94(2)	2.753(1)	162.2(16)
C2H2O4^ii^	0.95	2.59	3.500(1)	160
C5H5*Cg* ^iii^	0.95	2.75	3.534(1)	141

Symmetry codes: (i) 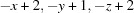; (ii) 
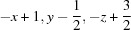
; (iii) 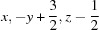.

**Table 2:** Experimental details

Crystal data	
Chemical formula	C_8_H_9_BO_4_
*M*_r_	179.96
Crystal system, space group	Monoclinic, *P*2_1_/*c*
Temperature (K)	100
*a*, *b*, *c* ()	11.2449(6), 12.0672(6), 6.8598(3)
()	105.121(1)
*V* (^3^)	898.61(8)
*Z*	4
Radiation type	Mo *K*
(mm^1^)	0.10
Crystal size (mm)	0.35 0.10 0.10

Data collection	
Diffractometer	Bruker *SMART* APEXII area detector
Absorption correction	Multi-scan (*SADABS*; Bruker, 2014 ▸)
*T*_min_, *T*_max_	0.706, 0.746
No. of measured, independent and observed [*I* > 2(*I*)] reflections	31992, 2056, 1872
*R*_int_	0.021
(sin /)_max_ (^1^)	0.649

Refinement	
*R*[*F* ^2^ > 2(*F* ^2^)], *wR*(*F* ^2^), *S*	0.032, 0.094, 1.10
No. of reflections	2056
No. of parameters	126
H-atom treatment	H atoms treated by a mixture of independent and constrained refinement
_max_, _min_ (e ^3^)	0.39, 0.22

Computer programs: *APEX2* and *SAINT-Plus* (Bruker, 2014 ▸), *SHELXT* (Sheldrick, 2015*a* ▸), *XP* in *SHELXTL* (Sheldrick, 2008 ▸), *SHELXL2014* (Sheldrick, 2015*b* ▸) and *publCIF* (Westrip, 2010 ▸).

## supplementary crystallographic information

### Crystal data

**Table:**

C_8_H_9_BO_4_	*F*(000) = 376
*M**_r_* = 179.96	*D*_x_ = 1.330 Mg m^−^^3^
Monoclinic, *P*2_1_/*c*	Mo *K*α radiation, λ = 0.71073 Å
*a* = 11.2449 (6) Å	Cell parameters from 9864 reflections
*b* = 12.0672 (6) Å	θ = 2.5–31.1°
*c* = 6.8598 (3) Å	µ = 0.10 mm^−^^1^
β = 105.121 (1)°	*T* = 100 K
*V* = 898.61 (8) Å^3^	Block, white
*Z* = 4	0.35 × 0.10 × 0.10 mm

### Data collection

**Table:**

Bruker SMART APEXII area-detector diffractometer	2056 independent reflections
Radiation source: sealed tube	1872 reflections with *I* > 2σ(*I*)
Detector resolution: 8.258 pixels mm^-1^	*R*_int_ = 0.021
φ and ω scans	θ_max_ = 27.5°, θ_min_ = 1.9°
Absorption correction: multi-scan (*SADABS*; Bruker, 2014)	*h* = −14→14
*T*_min_ = 0.706, *T*_max_ = 0.746	*k* = −15→15
31992 measured reflections	*l* = −8→8

### Refinement

**Table:**

Refinement on *F*^2^	0 restraints
Least-squares matrix: full	Hydrogen site location: mixed
*R*[*F*^2^ > 2σ(*F*^2^)] = 0.032	H atoms treated by a mixture of independent and constrained refinement
*wR*(*F*^2^) = 0.094	*w* = 1/[σ^2^(*F*_o_^2^) + (0.0494*P*)^2^ + 0.2551*P*] where *P* = (*F*_o_^2^ + 2*F*_c_^2^)/3
*S* = 1.10	(Δ/σ)_max_ < 0.001
2056 reflections	Δρ_max_ = 0.39 e Å^−^^3^
126 parameters	Δρ_min_ = −0.22 e Å^−^^3^

### Special details

**Table:**

Geometry. All e.s.d.'s (except the e.s.d. in the dihedral angle between two l.s. planes) are estimated using the full covariance matrix. The cell e.s.d.'s are taken into account individually in the estimation of e.s.d.'s in distances, angles and torsion angles; correlations between e.s.d.'s in cell parameters are only used when they are defined by crystal symmetry. An approximate (isotropic) treatment of cell e.s.d.'s is used for estimating e.s.d.'s involving l.s. planes.

### Fractional atomic coordinates and isotropic or equivalent isotropic displacement parameters (Å^2^)

**Table:**

	*x*	*y*	*z*	*U*_iso_*/*U*_eq_	
O1	0.90580 (7)	0.61792 (6)	0.84477 (13)	0.0258 (2)	
H1A	0.9797 (19)	0.5946 (15)	0.897 (3)	0.054 (5)*	
C1	0.67921 (8)	0.57954 (8)	0.78322 (14)	0.0163 (2)	
B1	0.81840 (10)	0.54762 (9)	0.87529 (16)	0.0185 (2)	
O2	0.85621 (7)	0.45309 (6)	0.98510 (12)	0.02287 (19)	
H2A	0.8020 (17)	0.4076 (14)	0.997 (3)	0.046 (4)*	
C2	0.58139 (9)	0.51156 (8)	0.80135 (14)	0.0166 (2)	
H2	0.5989	0.4434	0.8724	0.020*	
O3	0.21983 (6)	0.60624 (6)	0.54273 (12)	0.02136 (18)	
C3	0.45926 (9)	0.54182 (8)	0.71743 (14)	0.0161 (2)	
H3	0.3942	0.4945	0.7303	0.019*	
O4	0.27900 (6)	0.77238 (6)	0.45341 (11)	0.02041 (18)	
C4	0.43313 (8)	0.64218 (8)	0.61442 (13)	0.0153 (2)	
C5	0.52908 (9)	0.71113 (8)	0.59350 (14)	0.0169 (2)	
H5	0.5112	0.7793	0.5227	0.020*	
C6	0.65052 (9)	0.67947 (8)	0.67660 (15)	0.0177 (2)	
H6	0.7153	0.7263	0.6610	0.021*	
C7	0.30450 (8)	0.68109 (8)	0.52886 (14)	0.0163 (2)	
C8	0.09288 (9)	0.64329 (9)	0.47857 (19)	0.0271 (2)	
H8A	0.0381	0.5827	0.4945	0.041*	
H8B	0.0813	0.7065	0.5614	0.041*	
H8C	0.0735	0.6657	0.3364	0.041*	

### Atomic displacement parameters (Å^2^)

**Table:**

	*U*^11^	*U*^22^	*U*^33^	*U*^12^	*U*^13^	*U*^23^
O1	0.0150 (4)	0.0242 (4)	0.0365 (4)	−0.0005 (3)	0.0034 (3)	0.0100 (3)
C1	0.0156 (4)	0.0176 (5)	0.0155 (4)	0.0006 (3)	0.0036 (3)	−0.0014 (4)
B1	0.0160 (5)	0.0190 (5)	0.0199 (5)	0.0002 (4)	0.0035 (4)	0.0001 (4)
O2	0.0144 (3)	0.0211 (4)	0.0314 (4)	−0.0013 (3)	0.0029 (3)	0.0073 (3)
C2	0.0180 (5)	0.0156 (4)	0.0162 (4)	0.0017 (3)	0.0045 (3)	0.0007 (3)
O3	0.0147 (3)	0.0180 (4)	0.0306 (4)	0.0001 (3)	0.0046 (3)	0.0004 (3)
C3	0.0162 (4)	0.0160 (4)	0.0165 (4)	−0.0010 (3)	0.0051 (3)	−0.0017 (3)
O4	0.0190 (3)	0.0188 (4)	0.0232 (4)	0.0034 (3)	0.0051 (3)	0.0029 (3)
C4	0.0159 (4)	0.0162 (4)	0.0136 (4)	0.0014 (3)	0.0036 (3)	−0.0025 (3)
C5	0.0195 (5)	0.0156 (4)	0.0158 (4)	0.0011 (3)	0.0050 (3)	0.0010 (3)
C6	0.0166 (4)	0.0182 (5)	0.0188 (4)	−0.0015 (3)	0.0054 (3)	0.0007 (3)
C7	0.0172 (4)	0.0170 (4)	0.0148 (4)	0.0004 (3)	0.0045 (3)	−0.0031 (3)
C8	0.0147 (5)	0.0260 (5)	0.0396 (6)	0.0013 (4)	0.0053 (4)	0.0015 (5)

### Geometric parameters (Å, º)

**Table:**

O1—B1	1.3552 (13)	C3—C4	1.3942 (13)
O1—H1A	0.86 (2)	C3—H3	0.9500
C1—C2	1.4034 (13)	O4—C7	1.2191 (12)
C1—C6	1.4035 (13)	C4—C5	1.3992 (13)
C1—B1	1.5752 (14)	C4—C7	1.4880 (13)
B1—O2	1.3720 (13)	C5—C6	1.3891 (13)
O2—H2A	0.841 (18)	C5—H5	0.9500
C2—C3	1.3923 (13)	C6—H6	0.9500
C2—H2	0.9500	C8—H8A	0.9800
O3—C7	1.3336 (12)	C8—H8B	0.9800
O3—C8	1.4503 (12)	C8—H8C	0.9800
			
B1—O1—H1A	113.1 (12)	C5—C4—C7	117.91 (8)
C2—C1—C6	118.00 (8)	C6—C5—C4	119.75 (9)
C2—C1—B1	122.77 (8)	C6—C5—H5	120.1
C6—C1—B1	119.23 (8)	C4—C5—H5	120.1
O1—B1—O2	118.16 (9)	C5—C6—C1	121.19 (9)
O1—B1—C1	118.02 (9)	C5—C6—H6	119.4
O2—B1—C1	123.82 (9)	C1—C6—H6	119.4
B1—O2—H2A	118.0 (12)	O4—C7—O3	123.30 (9)
C3—C2—C1	121.43 (9)	O4—C7—C4	123.32 (9)
C3—C2—H2	119.3	O3—C7—C4	113.37 (8)
C1—C2—H2	119.3	O3—C8—H8A	109.5
C7—O3—C8	115.73 (8)	O3—C8—H8B	109.5
C2—C3—C4	119.48 (9)	H8A—C8—H8B	109.5
C2—C3—H3	120.3	O3—C8—H8C	109.5
C4—C3—H3	120.3	H8A—C8—H8C	109.5
C3—C4—C5	120.15 (9)	H8B—C8—H8C	109.5
C3—C4—C7	121.91 (8)		
			
C2—C1—B1—O1	178.38 (9)	C7—C4—C5—C6	178.03 (8)
C6—C1—B1—O1	−1.02 (14)	C4—C5—C6—C1	−0.52 (14)
C2—C1—B1—O2	−1.88 (15)	C2—C1—C6—C5	0.81 (14)
C6—C1—B1—O2	178.73 (9)	B1—C1—C6—C5	−179.77 (9)
C6—C1—C2—C3	−0.29 (14)	C8—O3—C7—O4	−5.45 (14)
B1—C1—C2—C3	−179.69 (9)	C8—O3—C7—C4	174.59 (8)
C1—C2—C3—C4	−0.52 (14)	C3—C4—C7—O4	173.53 (9)
C2—C3—C4—C5	0.82 (14)	C5—C4—C7—O4	−4.78 (14)
C2—C3—C4—C7	−177.45 (8)	C3—C4—C7—O3	−6.51 (13)
C3—C4—C5—C6	−0.31 (14)	C5—C4—C7—O3	175.18 (8)

### Hydrogen-bond geometry (Å, º)

Cg is the centroid of ring C1–C6.

**Table:**

*D*—H···*A*	*D*—H	H···*A*	*D*···*A*	*D*—H···*A*
O1—H1*A*···O2^i^	0.86 (2)	1.90 (2)	2.762 (1)	178.4 (18)
O2—H2*A*···O4^ii^	0.84 (2)	1.94 (2)	2.753 (1)	162.2 (16)
C2—H2···O4^ii^	0.95	2.59	3.500 (1)	160
C5—H5···*Cg*^iii^	0.95	2.75	3.534 (1)	141

Symmetry codes: (i) −*x*+2, −*y*+1, −*z*+2; (ii) −*x*+1, *y*−1/2, −*z*+3/2; (iii) *x*, −*y*+3/2, *z*−1/2.
